# A meta-analysis and systematic review of plant growth regulator use in blueberry production

**DOI:** 10.3389/fpls.2025.1632855

**Published:** 2025-08-20

**Authors:** Daniel Dick, Joshua VanderWeide

**Affiliations:** Department of Horticulture, Michigan State University, East Lansing, MI, United States

**Keywords:** vaccinium, gibberellic acid, cytokinin, auxin, abscisic ccid, ethylene, jasmonate, melatonin

## Abstract

Plant growth regulators (PGRs) include natural and synthetic plant phytohormones and other substances with the capacity to shape one or more aspects of plant growth and development at small concentrations. PGRs are commonly utilized in tree fruit and table grape production to reduce fruit set (thinning) and increase fruit size, coloration, and quality. However, use of PGRs in the production of berry crops, such as blueberry, is less common despite the abundance of production issues and the breadth of PGRs generally registered for fruit crops. This meta-analysis and systematic review discusses the past and current literature surrounding PGR use in blueberry production. First, we highlight the lack of PGRs registered and available to use in blueberry production relative to the increase in blueberry production value over the past decade. Next, we discuss the published literature on PGR use in blueberry species by production topic, including fruit set, berry mass and plant yield, ripening rate and harvest fruit quality, post-harvest fruit quality, and winter hardiness. Meta-analysis of qualifying PGR and production topic combinations revealed that gibberellic acid (GA_3_) and cytokinins (CKs) increase fruit set, CKs increase berry size, abscisic acid (ABA) and GA_3_ do not influence berry size, GA_3_ increases yield, and ABA does not enhance anthocyanin concentration. As global blueberry production continues to expand globally, PGR use will likely increase to address production issues and sustain production and fruit quality.

## 1 Introduction

Blueberry is a member of the *Vaccinium* L. genus and is currently the only economically important member of this genus besides cranberry (*Vaccinium macrocarpon* Aiton). Cultivated and wild blueberry production in North America encompasses highbush (*V. corymbosum* L.), rabbiteye (RE) (*V. virgatum syn. V. ashei*), and lowbush (*V. angustifolium* Aiton; also referred to as the wild blueberry) species. *V. corymbosum* includes northern highbush (NHB) as well as southern highbush (SHB), which is an interspecific hybrid between NHB and evergreen blueberry (*V. darrowii* Camp) (Boches et al., 2006). SHB is genetically indiscernible from NHB despite large differences in chilling requirements, cold hardiness, and production habits (Nishiyama et al., 2021). Rabbiteye blueberry (RE) is native to the Southeastern United States and is well-adapted to hot, humid climates, exhibiting greater stress tolerance compared to NHB and SHB. The production range of blueberry species in North America relates to their chilling requirements. NHB cultivars generally require more than 1000 chilling hours (ch) to satisfy dormancy (Kogan et al., 2023). NHB can be found across the northern U.S. and Canada, but large-scale production regions are centered in the Pacific Northwest regions of the U.S. and Canada, as well as the U.S. states Michigan and New Jersey. The chilling requirements for SHB are lower than NHB (250-600 ch), and this species is typically found in the Southeastern U.S. and California (Spiers et al., 2004). RE blueberries have chilling requirements of 350-700 ch, and are found in U.S. states Georgia, Alabama, North Carolina, Mississippi, and Texas (Spiers et al., 2004). Finally, lowbush (LB) have chilling requirements similar to NHB (>800-1000 ch) and are largely found in the U.S. state Maine and Canadian province Nova Scotia (Retamales and Hancock, 2018).

The popularity of blueberries with consumers has surged over the past two decades. From 2000 to 2019, consumption of fresh and processed blueberries (in millions of pounds) in the U.S. increased by 472% and 289%, respectively (USDA/ERS, 2021). This is largely due to the development of new cultivars with improved fruit quality and shelf life, which have been major goals of blueberry breeding programs in recent decades (Cappai et al., 2018; Edger et al., 2022). To meet consumer demand, commercial blueberry acreage continues to rise in the U.S. From 2005 to 2021, the acreage expanded by 58%, from 71,075 to 112,100 acres (USDA/NASS, 2022). The recent global expansion of blueberries into new production regions has resulted in the year-round production and availability of fruit, which has increased exposure of consumers to this crop (Bañados, 2009; Lobos and Hancock, 2015; Fang et al., 2020). Additionally, blueberries are known for their health properties and have gained the promotive label of a “superfruit” due to their high phenolic concentration and antioxidant activity, which has influenced consumer demand (Silva et al., 2020).

Dozens of blueberry cultivars are produced commercially in the U.S. The goal of breeding programs is to improve specific traits through new cultivar releases. However, both existing and new cultivars have production issues that affect the profitability of producers. These issues range from fruit quality to biotic and abiotic stress tolerance (Edger et al., 2022). In many cases, production issues are not realized until years after the establishment of new plantings. With these limitations in mind, horticultural practices are required to maximize each cultivar’s potential. In tree fruit and table grape production, plant growth regulators (PGRs) are simple, cost-effective, and common horticultural tools utilized to manage and mitigate cultivar-specific production issues (Greene, 1989; Bons and Kaur, 2020). PGRs include natural and synthetic plant phytohormones and other substances with the capacity to shape one or more aspects of plant growth and development at small concentrations. Many reviews have described the role of PGRs in fruit crop production (Rademacher, 2015; Hajam et al., 2017; Bisht et al., 2018; Ali, 2019; Baghel et al., 2019; Singh et al., 2025). One study reviewed PGR use in blueberry; however, this was limited to SHB and RE in the Southeastern United States (Nesmith, 2005). While NHB has similar production problems to SHB that can be addressed with PGRs, no review has summarized prior research.

PGRs can prevent or modulate in-season production problems and have been utilized in fruit crops since the 1930s. By promoting or inhibiting flowering, thinning excessive fruit, or synchronizing ripening, PGRs have allowed growers to maintain consistent yields and improve fruit quality year to year. Over time, research on PGRs has expanded beyond crop load regulation to address a broad range of production challenges, including fruit size, ripening uniformity, preharvest fruit drop, vegetative vigor, and even postharvest quality. These tools are particularly valuable because they offer chemical and physiological alternatives to labor-intensive practices like hand thinning or pruning. The specificity and timing of PGR applications make them adaptable to cultivar- and climate-specific issues, making them especially promising for perennial fruit crops like blueberry, where changes in management take years to manifest at scale. However, while the use of PGRs is well established in many crops, their adoption in blueberry remains limited, with fragmented research and few clear, generalized guidelines for applications.

## 2 Materials and methods

### 2.1 Data collection

A systematic literature search was performed to identify works on PGR use in blueberry in peer-reviewed scientific journals in April 2024. We used the following search phrases: (blueberry OR vaccinium) AND (PGR OR plant growth regulator) OR (abscisic acid OR ABA) OR (auxin OR NAA OR IAA OR IBA) OR (ethephon OR ethrel OR ethylene) OR (gibberellic acid OR GA OR gibberellin) OR (jasmonic acid OR JA OR jasmonate) OR (cytokinin OR CPPU OR BA OR 6-BA) OR (fruit set OR fruit retention) OR (yield OR berry size) OR (ripening rate) OR (harvest fruit quality) OR (post-harvest fruit quality) OR (winter hardiness OR cold tolerance OR frost resistance). Search terms were selected to represent each major class of PGRs and were expanded to include common synonyms, abbreviations, and related compounds within each class. Production topics were chosen based on known roles of PGRs in fruit crop production and previous topics of a PGR review in blueberry (Nesmith, 2005). The publication year of studies ranged from 1960 to 2024. Four search engines were utilized: Web of Science Core Collection, Google Scholar, Semantic Scholar, and Science.gov. Searches were replicated across all search engines. Google Scholar publications were screened for peer review manually, while other search engines pre-screened results automatically. Master’s theses and Doctoral dissertations as well as conference proceedings or publications were not included in the data analysis. The process of searching and selecting publications is outlined in the PRISMA (Preferred Reporting Items for Systematic Reviews and Meta-Analyses) flow diagram (**Supplementary Figure S1**). A total of 155 publications were identified that involved PGR use in blueberry. From these publications, 101 were excluded due to not measuring a comparable trait within our study scope, four were excluded as duplicate publications, and four were excluded due to not being peer reviewed. In total, 46 peer-reviewed studies were included in the meta-analysis.

### 2.2 Data curation

**Figure 1:**
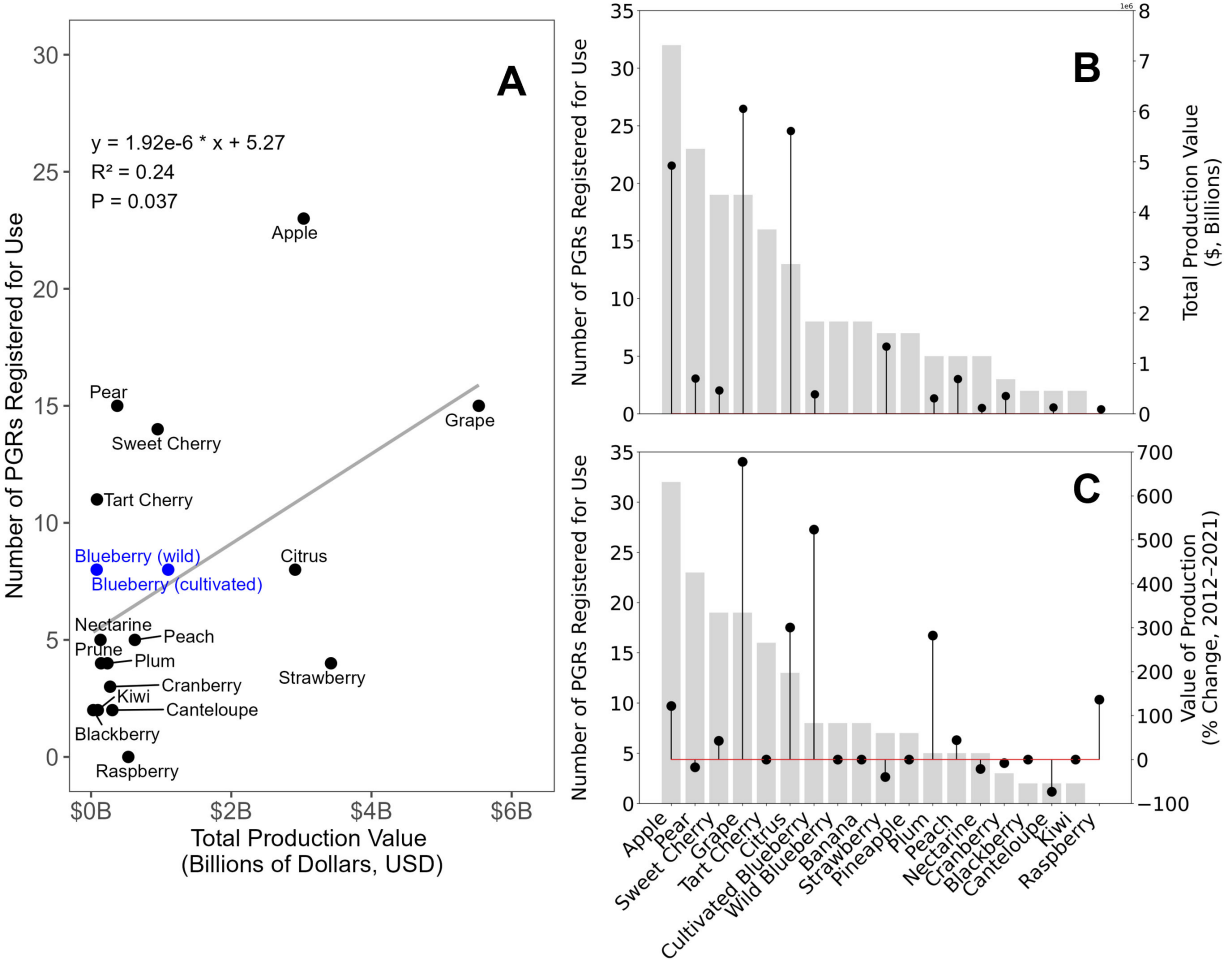
The **(A)** relationship between U.S.-grown fruit crop total production value ($, billions) in 2021 and the number of registered plant growth regulators for each crop, **(B)** the U.S.-grown total fruit crop production value ($, billions) in 2021 (black circles) and the number of PGRs registered for use in each crop (grey bars), and **(C)** the change (%) in U.S.-grown total fruit crop production value ($, billions) from 2012 to 2021 (black circles) and the number of PGRs registered for use in each crop (grey bars).

Data curation was performed similarly to our previous meta-analyses (VanderWeide et al., 2021, 2022a, 2024). Publications were maintained for further statistical analysis if they contained data categorized under the following self-defined physiological topics: fruit set, berry mass and plant yield, ripening rate and harvest fruit quality, post-harvest fruit quality, and winter hardiness. Publications that did not provide quantitative measurements related to these categories were excluded from further analysis. The exclusion of publications to fit these criteria resulted in 48 studies. For each publication, in the case that desired data were only present in figures, ImageJ software (Version 1.51e, Schneider et al., 2012) was utilized to extract data points when the treatments from the respective publication were distinguishable. Across all production topics, the units of each measure were standardized to a common unit and a conversion was made when necessary. The number of observations (n) by 1) PGR, 2) blueberry species, and 3) production topic was summed across all studies. Results were tabulated and are presented as **Figure 1**. To extract mean values and variance measures within and across studies, all reported values for the same PGR within a study were averaged to generate a single representative value per study per hormone, while confidence in the mean was weighed according to n. If multiple time points were available within a study (e.g. multiple harvest dates), values were averaged across all relevant sampling points to ensure comparability across studies. Variance measures (e.g., standard deviation or standard error) were extracted from all publications. In publications where only graphs contained the standard deviation or standard error data, ImageJ was used to estimate confidence bands. Publications differed in their reporting of blueberry growth stages, so reported growth stages were converted to the BBCH scale, according to (Wichura et al., 2024).

### 2.3 Statistical analysis

Meta analyses procedures were performed according to (Schwarzer et al., 2015) and used the ‘meta’ package in R version 4.2.1 (Schwarzer, 2006). Data for the analysis included numerical information such as means, standard deviations, and sample sizes for both treatment groups (e.g., untreated control, PGR-treated). Additionally, subgroup classifications, including hormone type (e.g. GA_3_), and specific production topic (e.g. fruit set), were recorded to allow for separate analyses of distinct factors. A continuous statistical method was employed using the ‘metacont’ function from the ‘meta’ package (Schwarzer, 2006), which employs inverse variance weighting for the analysis, ensuring that studies with larger sample sizes (observations) or more precise estimates contribute more to the overall effect, as weighted by n. Meta-analyses were conducted only when four or more studies were available for a given comparison. This threshold was chosen to ensure both statistical reliability and interpretive value. From a statistical standpoint, fewer than four studies provide limited degrees of freedom for estimating between-study variance (τ²) and calculating heterogeneity measures such as the Q statistic or I² (Schwarzer et al., 2015).

Considering the substantial variability across studies in species, cultivars, experimental designs, and protocols, a random-effects model was chosen to account for between-study heterogeneity. A random effects model was used to determine whether variance between studies out-competed the variance within studies, providing confidence that differences between studies could be attributed to actual differences in responses due to external factors, rather than an error in the methodology. The DerSimonian-Laird (DL) method was used to estimate heterogeneity ( τ ²), which provides a robust framework for combining results from diverse studies (Schwarzer et al., 2015). Mean difference (MD) was chosen as the primary effect size metric for the forest plots, as the measurement scales were consistent across studies. This allowed the overall effect to be expressed in a unit-dependent and meaningful manner. Analyses were also conducted without standardizing units (Standardized Mean Difference, SMD), but in all cases, MD yielded higher confidence in estimating between-study variance, thus MD was chosen as the proper testing methodology. Weighted means, effect sizes, and 95% confidence intervals (CIs) were calculated through ‘metacont’ and summarized in the analysis.

Forest plots were generated for each meta-analysis, providing a comprehensive visual representation of individual study results and overall effect sizes. The forest plots were designed to include detailed heterogeneity statistics, including Cochran’s Q test, τ², and I², to assess the degree of variability among studies. Heterogeneity was considered significant when I² exceeded 75% or when the *p*-value of the Q test was less than 0.05, in accordance with (Higgins, 2003), which are widely accepted standards in meta-analytic methodology. Additionally, significance was indicated when the 95% CI of an effect size did not overlap the null effect line. The overall effect size was evaluated using Z-scores and *p*-values to determine statistical significance. By providing both Z-scores and mean differences (MD), we present two comparative measures: Z-scores, which express the mean difference in terms of standard deviation units, and MD, which represents the absolute mean difference in the original measurement units.

## 3 Results and discussion

### 3.1 Number of registered PGRs by fruit crop

In the U.S., under the Federal Insecticide, Fungicide, and Rodenticide Act (FIFRA), most PGRs are considered pesticides and must be approved by the U.S. EPA before they can be used in the production of a specific crop. This requires a significant time commitment and financial investment from manufacturers to meet regulatory standards (1996). As a result, crops with higher production value may attract more investment into PGR development, increasing the number of registered PGRs, while lower-value crops may see fewer PGR registrations due to the costs involved.

We were interested to understand whether the production value of fruit crops in the U.S. predicted the number of PGRs registered for that crop. The total U.S. production value of a particular fruit crop did correlate significantly (*p=*0.037) with the number of PGRs registered for that crop (**Figure 1A**). However, some exceptions were noted. As of 2021, blueberry (cultivated and wild) had a total production value of $834 million and seven PGRs were registered for use in the crop (USDA/NASS, 2022), (**Figure 1B**). Fruit crops with a similar number of registered PGRs as blueberry, such as strawberry ($2.9 billion; six registered PGRs) and pineapple ($10 million; six registered PGRs) had vastly different production values compared to blueberry. Additionally, multiple fruit crops that had a similar production value to blueberry, such as sweet cherry ($891 million; 18 registered PGRs) and pear ($519 million; 23 registered PGRs) had nearly threefold more PGRs registered. Sweet cherry fruit are prone to cracking and poor color development at harvest (Zhang and Whiting, 2011; Correia et al., 2018). Many of the PGRs registered for use in sweet cherry were developed to mitigate these issues. Tree fruit crops such as apple, pear, and peach have the capacity to set more fruit than can be ripened to maturity (Bangerth, 2000; Jackson, 2003; Westwood, 2009). Thus, many of the PGRs registered in these crops have been developed to thin flowers and fruit to modulate crop load, as well as reduce the magnitude of alternate bearing across seasons, the phenomenon where fruit set of one year influences the capacity of the next.

Over the past decade, the production value of blueberry in the U.S. increased by 518%, second only to grape at 680% (**Figure 1C**). The increase in production value is largely due to the drastic increase in blueberry acreage throughout the U.S. in the same time frame. Blueberry is among the fastest-growing fruit crops in the U.S.; production has increased by 51% in the past decade. Globally, blueberry production has doubled in the last decade. Expansion of acreage is occurring in both established and new production regions (NeSmith and Krewer, 1997; Bañados, 2009; Lobos and Hancock, 2015), and different growing regions offer different production challenges. Traditional blueberry management techniques may not be optimal in these regions, and PGRs have the capacity to offer regionally adaptable solutions to overcome physiological, abiotic, and biotic challenges.

**Figure 2:**
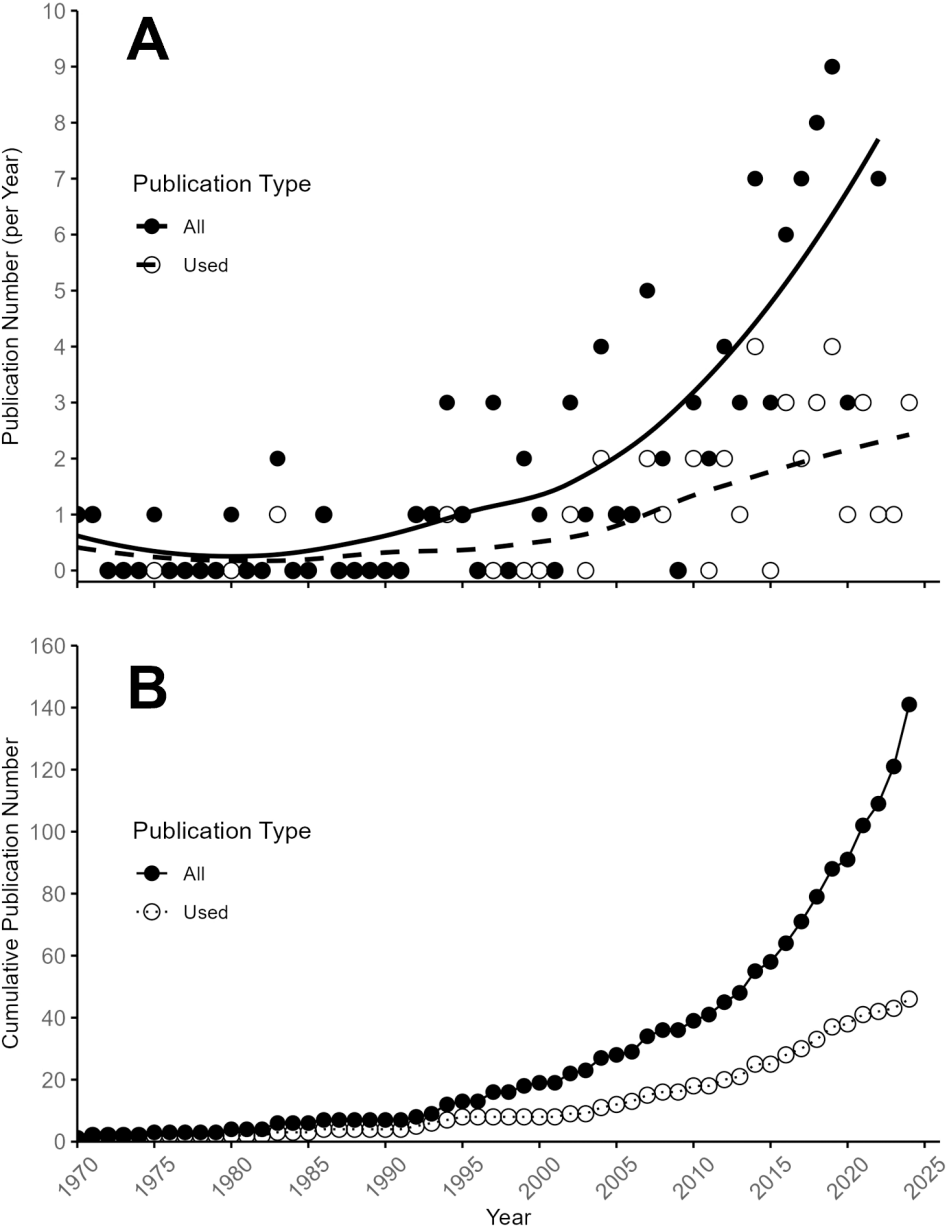
Trends in blueberry PGR publication count over time. **(A)** Annual number of publications, with solid black circles representing all publications and open circles indicating those included in the meta-analysis. A generalized mixed model illustrates the trend in publication growth. **(B)** Cumulative number of publications over time, distinguished between all published studies and those qualifying for the meta-analysis.

We analyzed the trend in PGR research in blueberry by publication year in **Figure 2**, which indicated that the total number of publications per year increased substantially in the 1990s, and has seen a significant increase in the last decade. The number of publications that did not include comparable metrics for use in our meta-analysis increased at a higher rate per year than that of publications that did, highlighting that more recent publications attempt to answer mechanistic, genetic, or cultural questions rather than a production-related component (**Figure 2A**). The total number of publications included in the meta-analysis (**Figure 2B**) indicated that most publications included were published in the 1990s through the early 2020s, with few publications included either before 1990 or after 2020.

### 3.2 Total PGR studies by year, hormone, and production topic

**Figure 3:**
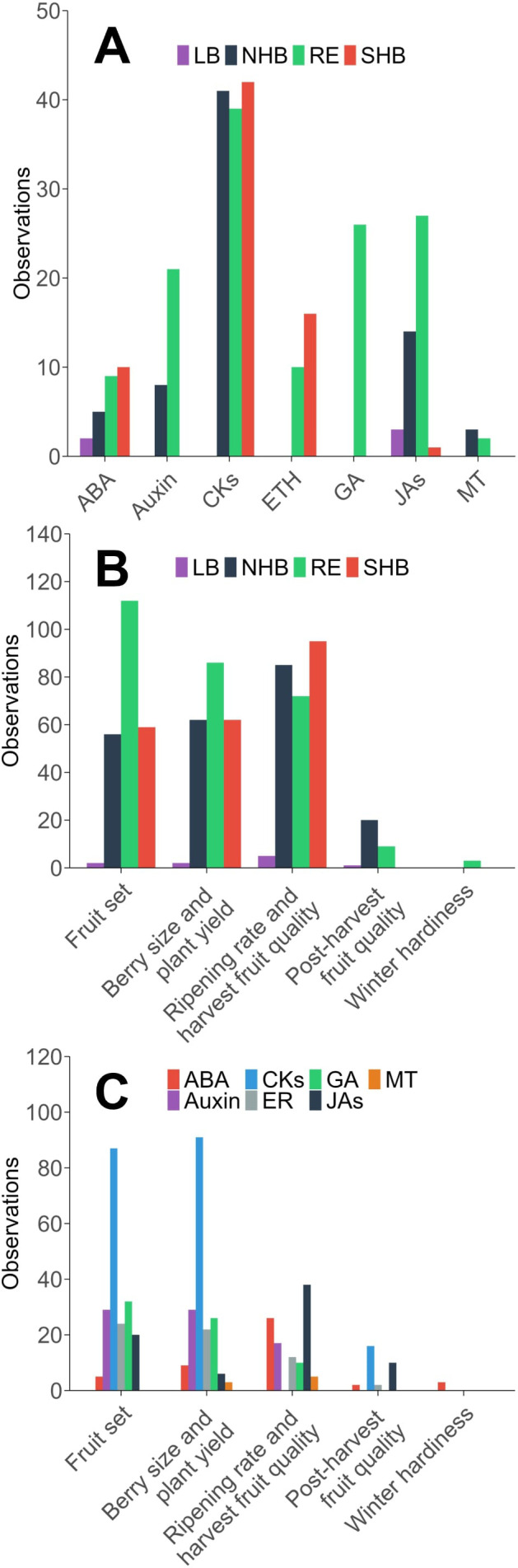
The number of observations of plant growth regulators in the scientific literature in blueberry, separated by the **(A)** phytohormone active ingredient and blueberry species, **(B)** production topic and blueberry species, and **(C)** production topic and phytohormone active ingredient. NHB, northern highbush; SHB, southern highbush; RE, rabbiteye; LB, lowbush; ABA, abscisic acid; BR, brassinosteroids; CKs, cytokinin-based phytohormones; ER, ethylene-based phytohormones; GA, gibberellic acid; JAs, jasmonates; MT, melatonin.

Literature observations were primarily grouped into the categories of “fruit set,” “berry size and plant yield,” and “ripening rate and harvest fruit quality.” Across observations, CKs had the most observations with 87 for “fruit set,” 91 for “berry size and yield,” and 135 for “ripening rate and harvest quality” (**Figure 3C**). This is despite the first CK studies being published after the initial studies on GA_3_. CK’s role in cell division, expansion, and delaying senescence likely explains the emphasis on this phytohormone. CK’s limited presence in post-harvest studies (16 observations) suggests less focus on CK’s for post-harvest storage or fruit quality. GA_3_ was most frequently studied in relation to “fruit set” (32 observations) and “berry size and plant yield” (26 observations), aligning with its well-established role in cell expansion, fruit elongation, and parthenocarpic fruit development (**Figure 3C**). While GA_3_ can effectively stimulate fruit set in RE and SHB blueberries, its tendency to produce smaller, seedless berries has prompted research into alternative or supplemental treatments with CKs (NeSmith, 2002). Interestingly, GA_3_ had no recorded observations for “ripening rate” or “post-harvest fruit quality,” reflecting a lack of research into their potential role in late-stage fruit development. Research on auxin followed a pattern similar to that of CKs and GA_3_, with a major focus on “fruit set” (29 observations) and “berry size and plant yield” (29 observations), but low representation in “ripening rate and harvest quality” (17 observations) and “post-harvest fruit quality” (0 observations) (**Figure 3C**). Auxins play a fundamental role in early fruit development by stimulating ovary growth and retention, which explains their frequent use in improving “fruit set” and “berry size and plant yield” (Liu et al., 2022b). However, their absence in ripening and post-harvest studies suggests that auxin-based PGRs have not been widely explored for improving storage characteristics or delaying senescence in blueberries. MeJA research has mainly focused on “ripening rate and harvest quality” (38 observations), with very little research on other topics. This may be because MeJA has been shown to increase secondary metabolites in other fruits (VanderWeide et al., 2023). Meanwhile research into “post-harvest fruit quality” is less studied. Ethylene-related (ER) PGR studies were grouped primarily into “fruit set” (24 observations), and “berry size and plant yield” (22 observations), with less attention for other production topics (**Figure 3C**). This is most likely because early ER research found ethylene promoting compounds decreased firmness and storability. The majority of modern ER research has focused more on ethylene inhibitors and the genetics of ripening; however, there were not enough studies to warrant meta-analysis. MT is the most recently discovered and least studied class of PGR and research has only been carried out on the topics of “ripening rate and harvest fruit quality” (5 observations) and “berry size and plant yield” (3 observations).

### 3.3 PGR use in blueberry according to production trait

#### 3.3.1 Fruit set

Fruit set, in horticultural terms, refers to the percentage of flowers that develop into mature fruit, though botanically it describes the transition from flower to initial fruit following fertilization. Here, we refer to the horticultural definition when discussing PGR effects. Fruit set in blueberry is highly dependent on environmental factors such as temperature, cultural practices such as pruning, as well as pollinator activity to ensure optimal development (Dogterom et al., 2000; Bieniasz, 2007; DeVetter et al., 2022). Reduced pollinator presence or unfavorable weather during the flowering period can lead to incomplete fruit set, affecting overall yield. RE suffers from naturally poor fruit set – typically less than 30% – while SHB and NHB typically set 60-80% of flowers and are capable of setting 100% when resources are not limited (NeSmith and Krewer, 1997; Dogterom et al., 2000). Blueberry does not experience reductions in berry size at high fruit set percentages as is the case in tree fruit crops such as apple or peach (Dennis, 2000). Rather, greater pollination and fruit set in blueberry produces larger fruit (NeSmith and Krewer, 1997; DeVetter et al., 2022). Given this, fruit set is a critical determinant of yield in blueberry.

**Figure 4:**
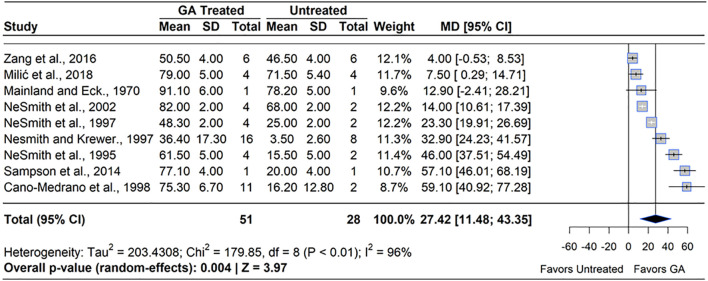
Forest plot of the mean difference (MD) in fruit set percentage between gibberellin (GA_3_)-treated and untreated blueberry plants. Each grey square represents an individual study’s effect size, with square size proportional to the study’s weight. The black diamond represents the overall mean difference, with its width reflecting the 95% confidence interval. The overall effect is considered significant if either value representing the 95% CI does not cross zero. Heterogeneity statistics are presented below the total estimate, and the bolded *p*-value also indicates overall effect.

GA_3_ has been widely studied for its role in enhancing fruit set by mimicking or amplifying the hormonal signals of pollination. Exogenous GA_3_ has been shown to substitute downstream signals of pollination, which retains more fruit on the plant than pollination signals alone (Hu et al., 2023). GA_3_ applications showed an overall positive impact on fruit set in blueberry. A meta-analysis of nine studies revealed that GA_3_ treatment significantly increased fruit set compared to untreated controls (MD = 27.42%, 95% CI: [11.48, 43.35], *p*=0.004) (**Figure 4**). Of the nine studies included, seven reported statistically significant increases in fruit set, with effects ranging from an 11.0% increase (Milić et al., 2018) to a 365.0% increase over the control (Cano-Medrano and Darnell, 1998). However, the effect of GA_3_ was not consistent across species. In RE, five studies reported a significant increase in fruit set under GA_3_ (100 – 484 ppm) application applied at 50-100% full bloom (BBCH 65 – 68). NHB showed a positive response in one study (Mainland and Eck, 1971) but no effect in another (Milić et al., 2018), while no studies on southern highbush blueberry (SHB) were identified. This stronger GA_3_ response in RE may partly reflect the inherently lower baseline fruit set commonly observed in this species, suggesting greater potential for improvement under PGR treatment. The greatest response to GA_3_ in RE has been shown to be between 0 and 14 DAFB. Heterogeneity statistics (I²=96%, τ^2^ = 203.43) indicated substantial variability of effect sizes between studies (Schwarzer et al., 2015), suggesting that factors such as cultivar, environmental conditions, and application technique played a significant role in determining GA_3_ efficacy.

**Figure 5:**
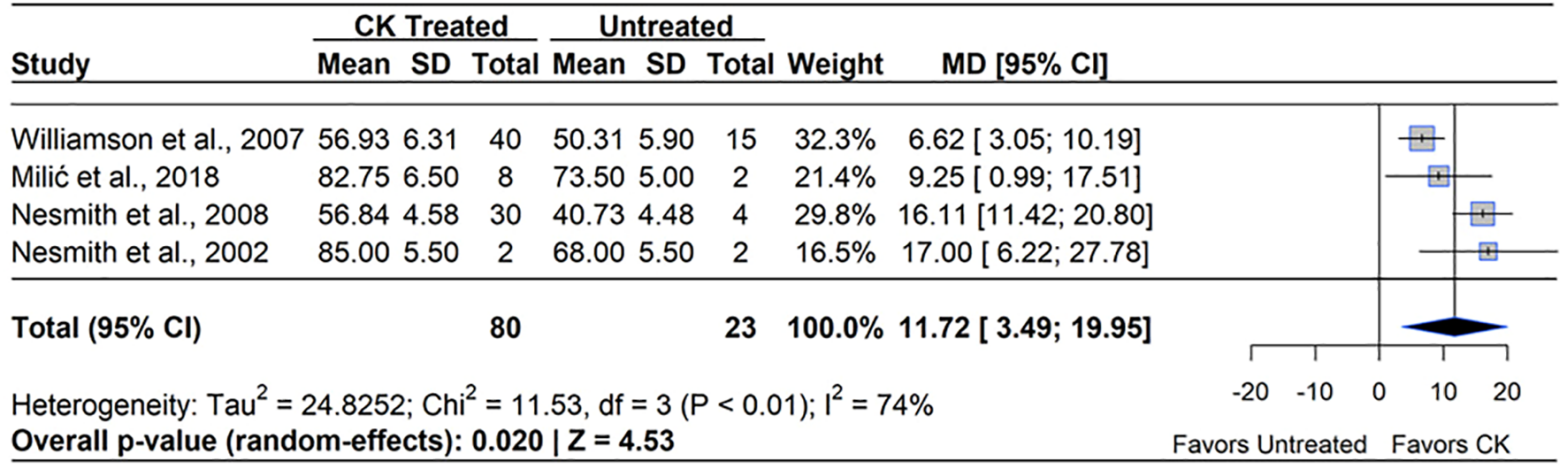
Forest plot of the mean difference (MD) in fruit set percentage between cytokinin (CK)-treated and untreated blueberry plants. Each grey square represents an individual study’s effect size, with square size proportional to the study’s weight. The black diamond represents the overall mean difference, with its width reflecting the 95% confidence interval. The overall effect is considered significant if either value representing the 95% CI does not cross zero. Heterogeneity statistics are presented below the total estimate, and the bolded *p*-value also indicates overall effect.

CK (all applied as *N*-(2-Chloropyridin-4-yl)-*N*′-phenylurea, Forchlorfenuron, CPPU) applications also showed a positive impact on fruit set in blueberry (MD = 11.72%, 95% CI: [3.49, 19.95], *p*=0.020). Across the four studies included in the meta-analysis, there were a total of 80 total application observations, all of which reported significant improvements in fruit set with application concentrations ranging from (10 – 20 ppm) (**Figure 5**). The treatment effect ranged from a 6.62% (Williamson and NeSmith, 2007) to a 17.0% increase compared to the untreated control (NeSmith, 2002). The magnitude of improvement varied across studies, with the greatest significant increase observed in RE cultivars (NeSmith, 2002, 2008). Meanwhile, NHB and SHB cultivars showed more moderate but significant effects (Williamson and NeSmith, 2007; Milić et al., 2018). All studies that showed increases in fruit set applied CPPU after initial flowering, with the greatest impact on fruit set shown with applications made between 7 – 14 DAFB. Heterogeneity statistics indicated moderate variability between studies (I²=74%, τ^2^ = 24.83), suggesting that species differences, application timing, and environmental conditions influenced the response to CK. Notably, within-study variability was greater than across-study variability, supporting the concept that differences in response were due to true biological variability rather than methodological inconsistencies. Despite this variability, the overall CK treatment effect was statistically significant (*p*=0.020, Z=4.53), supporting the use of CPPU as an effective tool for enhancing fruit set in blueberry.

NeSmith evaluated combined GA_3_ and CK (CPPU) treatments together over two years, under the hypothesis that GA_3_ would primarily enhancing pollen tube growth and fruit initiation, and CPPU would promote sustained fruit retention and development (NeSmith, 2002). While untreated control (open pollinated) treatments averaged 68% fruit set, GA_3_ (200 ppm), CPPU (10 ppm) and GA_3_+CPPU (200 ppm GA_3_ + 200 ppm CPPU) averaged 82%, 89%, and 92% fruit set, respectively. The slight additive effect observed when GA_3_ and CPPU were combined was also significantly greater than the control, but not against each hormone individually.

#### 3.3.2 Berry size and plant yield

Maximizing berry size (mass and diameter) and yield is a preeminent goal for blueberry producers. Berry size is largely under genetic control but can also be strongly modulated by environmental factors and pollination (Finn et al., 2003). In general, seed number per berry is positively correlated with berry mass and diameter (Brewer and Dobson, 1969; Benjamin and Winfree, 2014), and weather and pollination heavily influence the seed number per berry (Dogterom et al., 2000). For most blueberry cultivars, berry size is often greatest in the first ripening berries, and the size of berries that develop and ripen afterward gradually decreases (Ehlenfeldt, 2005; Milić et al., 2018). Large blueberries are preferred by consumers compared to small berries, and producers stand to benefit by providing larger berries for the market (Saftner et al., 2008). Calculating yield is complex and must take into account the number of canes per plant and berries per cane, as well as fruit set and plant age, which makes it difficult to accurately measure yield (Siefker and Hancock, 1986). Much research has been conducted using PGRs to improve blueberry mass and yield; however, due to the difficulty in estimating yield, this parameter was not always assessed in studies that measured berry mass.

**Figure 6:**
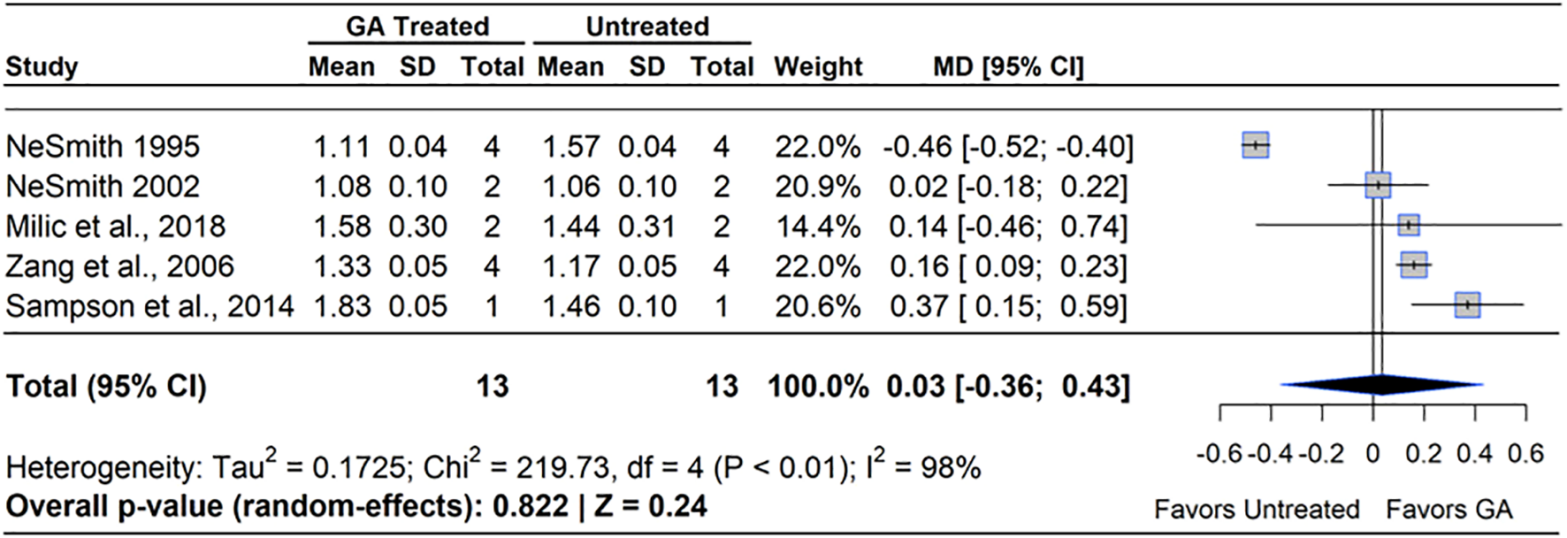
Forest plot of the mean difference (MD) in berry mass (g) between gibberellin (GA_3_)-treated and untreated blueberry plants. Each grey square represents an individual study’s effect size, with square size proportional to the study’s weight. The black diamond represents the overall mean difference, with its width reflecting the 95% confidence interval. The overall effect is considered significant if either value representing the 95% CI does not cross zero. Heterogeneity statistics are presented below the total estimate, and the bolded *p*-value also indicates overall effect.

The role of GA_3_ in fruit set was first discovered in the 1960s and numerous agricultural trials beginning in the early 1970s. However, GA_3_ treated fruit were shown to not have the same internal structure as pollinated berries and had a flatter shape due to lack of seed pockets (Mainland and Eck, 1971). A meta-analysis of the five qualifying studies revealed that GA_3_ treatment had no effect on berry mass compared to untreated controls (MD=3.00%, 95% CI: [-0.36, 0.43], *p*=0.240), (**Figure 6**). Two of the five studies reported increased berry mass under GA_3_ application, with improvements spanning from 18.5% (Zang et al., 2016) to 39% (Sampson et al., 2014). However, one study in blueberry reported that GA_3_ significantly decreased berry mass, with the reduction ranging from 45-50% (NeSmith et al., 1995). Heterogeneity statistics (I²=98%, τ^2^ = 0.17) indicated substantial between-study variability (Schwarzer et al., 2015), suggesting that factors such as cultivar, environmental conditions, and application technique played a significant role in determining GA_3_ efficacy. In RE, a reduction of berry mass was more pronounced than in NHB or SHB, as GA_3_ set more fruit in these RE studies.

**Figure 7:**
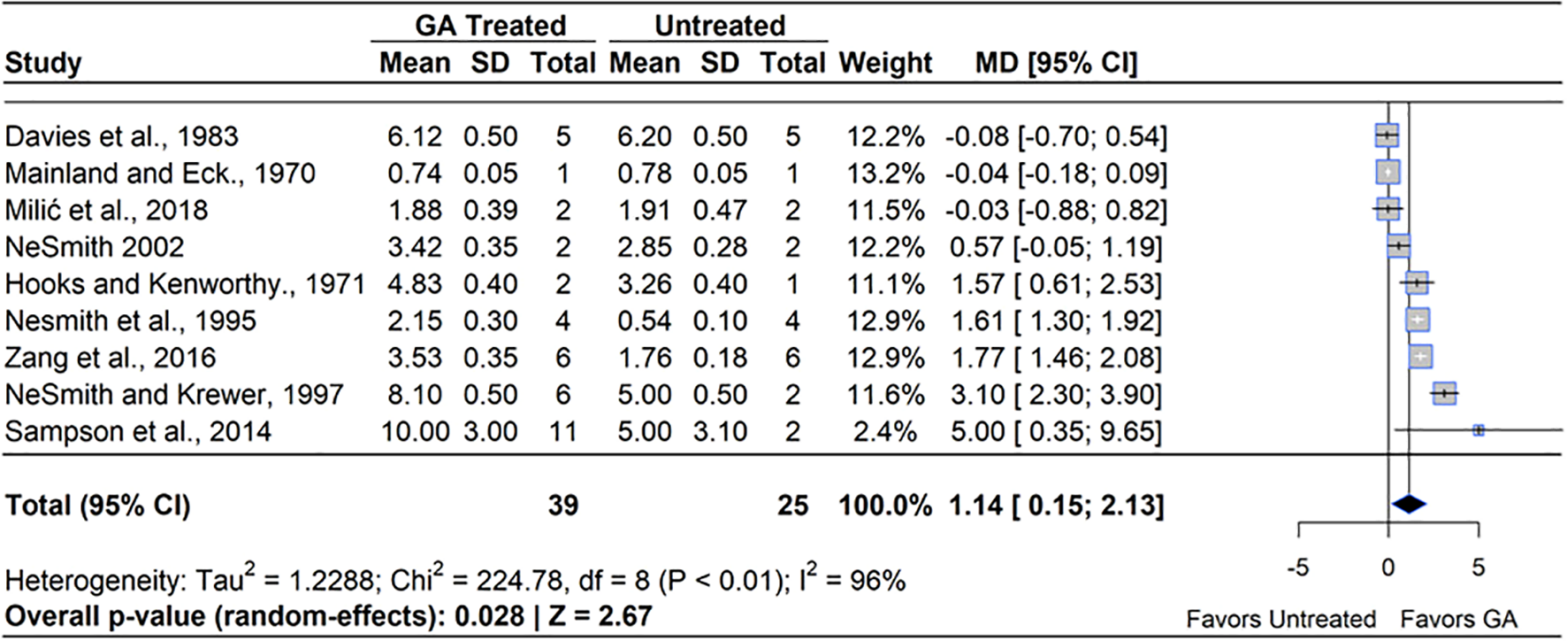
Forest plot of the mean difference (MD) in fruit yield (kg/plant) between gibberellin (GA_3_)-treated and untreated blueberry plants. Each grey square represents an individual study’s effect size, with square size proportional to the study’s weight. The black diamond represents the overall mean difference, with its width reflecting the 95% confidence interval. The overall effect is considered significant if either value representing the 95% CI does not cross zero. Heterogeneity statistics are presented below the total estimate, and the bolded *p*-value also indicates overall effect.

A meta-analysis of the nine qualifying studies revealed that GA_3_ treatment had a positive effect on yield compared to untreated controls (MD=49.51%, 95% CI: [0.15, 2.13], *p*=0.028), (**Figure 7**). Out of the nine studies included, six studies reported a significantly higher yield under GA_3_ treatment, which ranged from 20% to 100%, while three reported statistically significant decreases in yield, which ranged from 1.3% to 5.3%. Heterogeneity statistics (I²=96%, τ^2^ = 1.23) indicated substantial between-study variability (Schwarzer et al., 2015), which can be attributed to differences in induced fruit set and the yield that likely arose from the difficulty in yield calculation. Given that GA_3_ increased fruit set, but not berry mass, the greater yield can be attributed to higher fruit set. Most studies measured the yield of only a few plants, which may contribute to the heterogeneity of the study effects. The greatest increase in yield was experienced in RE, while the single study in NHB did not see a significant increase in yield compared to the control.

**Figure 8:**
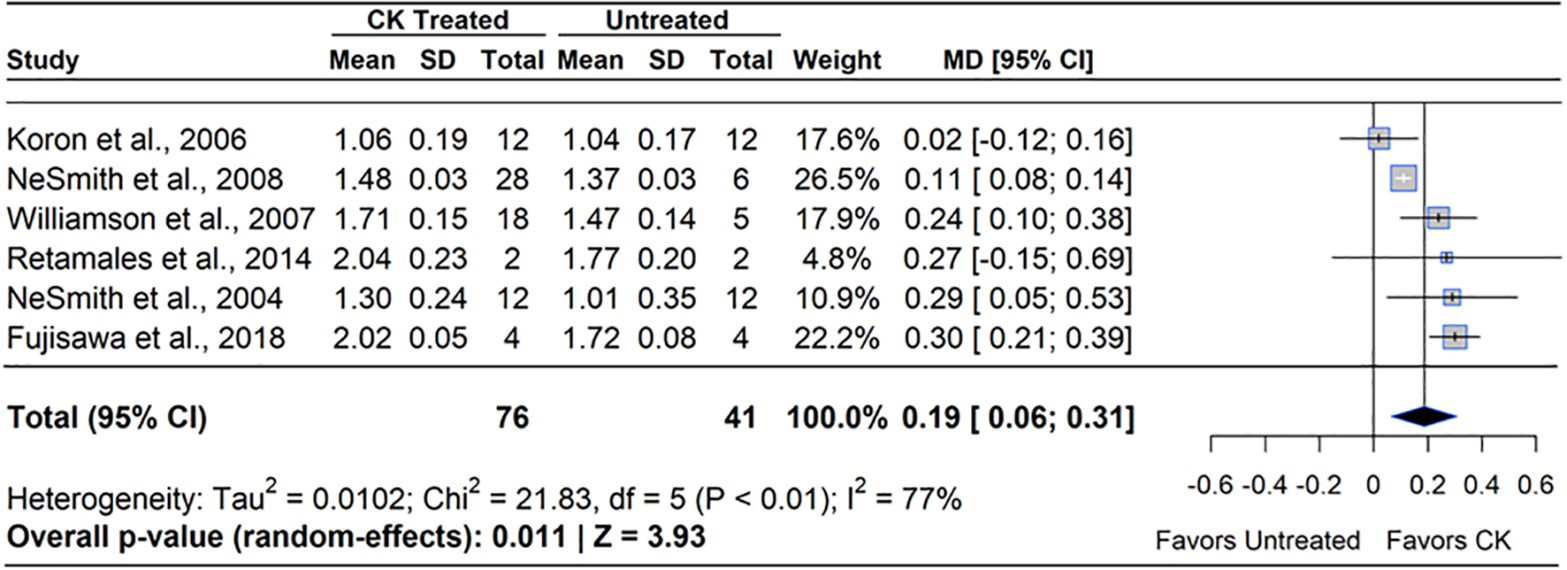
Forest plot of the mean difference (MD) in berry mass (g) between cytokinin (CK)-treated and untreated blueberry plants. Each grey square represents an individual study’s effect size, with square size proportional to the study’s weight. The black diamond represents the overall mean difference, with its width reflecting the 95% confidence interval. The overall effect is considered significant if either value representing the 95% CI does not cross zero. Heterogeneity statistics are presented below the total estimate, and the bolded *p*-value also indicates overall effect.

CK (CPPU) increased blueberry mass by an average of 14% (**Figure 8**). Of the six studies included, four reported a gain in berry mass under the CPPU treatment, ranging from 8-16% greater than the untreated control (MD=14.29%, 95% CI: [3.82, 17.66], *p*=0.011). Studies noted a band of sensitivity to CPPU, where concentrations higher than 10 ppm or applications applied during full bloom (FB) caused flower and leaf injury (Williamson and NeSmith, 2007). The worst CPPU injury was observed when CPPU was applied twice or more; new growth was triggered by the first application and caused floral necrosis with the second or further applications. All studies that reported an increase in berry mass and no injury to flowers or berries applied CPPU at least one week after FB, when berries were rapidly gaining mass (BBCH 71 - 72).

**Figure 9:**
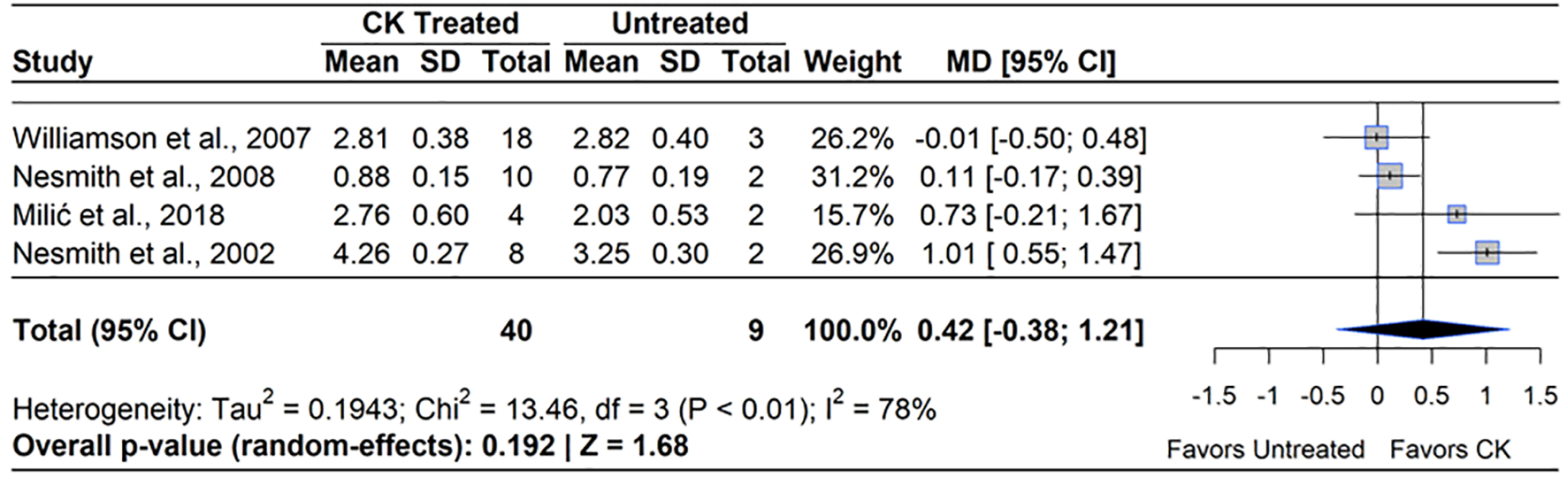
Forest plot of the mean difference (MD) in fruit yield (kg/plant) between cytokinin (CK)-treated and untreated blueberry plants. Each grey square represents an individual study’s effect size, with square size proportional to the study’s weight. The black diamond represents the overall mean difference, with its width reflecting the 95% confidence interval. The overall effect is considered significant if either value representing the 95% CI does not cross zero. Heterogeneity statistics are presented below the total estimate, and the bolded *p*-value also indicates overall effect.

Although CPPU significantly increased berry mass, only four studies reported effects on yield. Three of the studies were conducted on RE, while one was conducted on SHB. Of these four studies, two noted an increase in yield, ranging from 30-45% (NeSmith, 2002, 2008). A meta-analysis of the four qualifying studies indicated no effect on yield (MD=21.26%, 95% CI: [-0.38, 1.21], *p*=0.192) (**Figure 9**). Heterogeneity statistics (I²=78%, τ^2^ = 0.19) indicated high between-study variability (Schwarzer et al., 2015). The two studies which found no change in yield from control used fewer plants to estimate yield, which may have not been representative of treatment effects (Williamson and NeSmith, 2007; NeSmith, 2008).

In some seasons, the combined use of GA_3_ (200 ppm) and CPPU (10 ppm) showed more promising results on yield than the use of either hormone individually. NeSmith (2002) applied the combination of GA_3_ and CPPU for two years, and in both years, only CPPU increased berry mass by an average of 27% over the untreated control. In a season with a typical fruit set, CPPU alone increased yield through a higher fruit set and berry mass. In a season with naturally low fruit set, CPPU and GA_3_+CPPU increased yield due to an improvement of both fruit set and berry mass. In the low fruit set season, GA_3_+CPPU significantly improved yield compared to CPPU alone by an additional 18% because GA_3_ induced 11% higher fruit set than CPPU. This result may be due to a greater modification of genes involved in fruit set, cell division, and cell expansion using both hormones (Kamínek, 2015; Zang et al., 2016).

**Figure 10:**
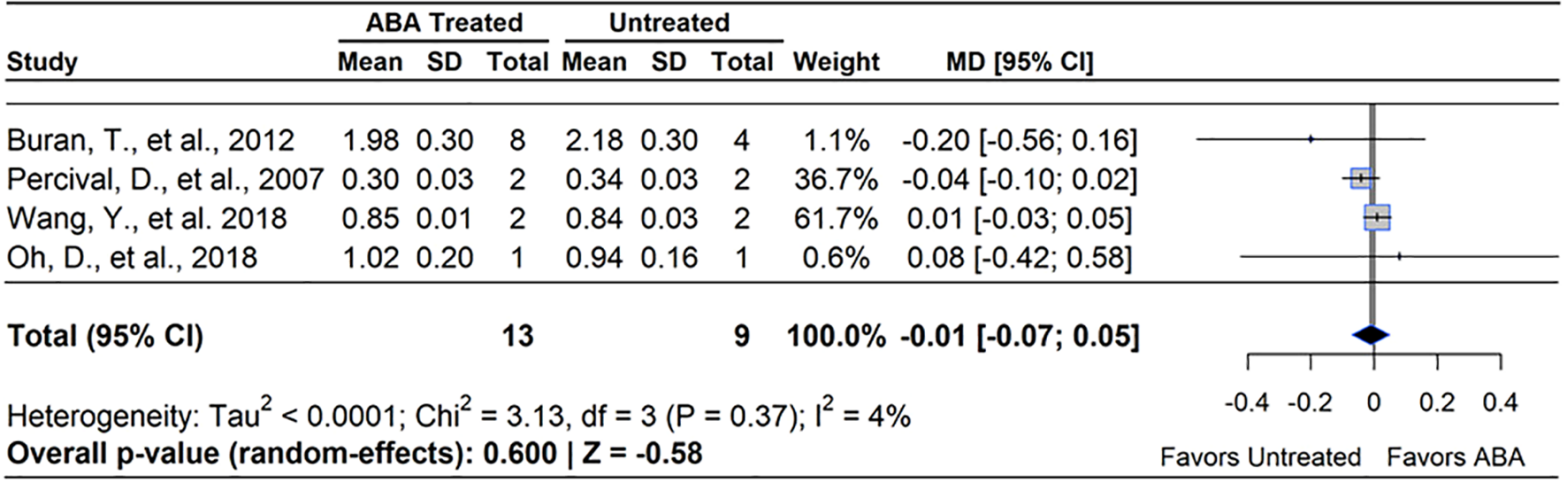
Forest plot of the mean difference (MD) in berry mass (g) between abscisic acid (ABA)-treated and untreated blueberry plants. Each grey square represents an individual study’s effect size, with square size proportional to the study’s weight. The black diamond represents the overall mean difference, with its width reflecting the 95% confidence interval. The overall effect is considered significant if either value representing the 95% CI does not cross zero. Heterogeneity statistics are presented below the total estimate, and the bolded *p*-value also indicates overall effect.

According to a meta-analysis of four studies, ABA had a null effect on berry mass (MD= -3.44%, 95% CI: [-0.07, 0.05], *p*=0.600), (**Figure 10**). Heterogeneity statistics (I²=4%, τ^2<^0.0001) indicated extremely low between-study variability (Schwarzer et al., 2015), although one study accounted for >60% of the weight among studies, which raises concerns about the overall power of the result (Wang et al., 2018). This suggests that only 4% of the observed variation in effect sizes was due to true heterogeneity, with the remaining 96% likely attributable to random sampling error rather than real differences between studies. Concentrations of ABA application in the studies ranged from 20 – 1000 ppm.

Auxins are known for their role in apical dominance of growing shoot and root tips, as well as in cell division and expansion (Crane, 1964). Initial studies studying auxin effects on berry size hypothesized that cell division and expansion would also be increased in developing fruit. The naturally occurring auxin Indole-3-acetic acid (IAA) (1000 ppm) was initially shown to improve berry mass in LB blueberry; however, this was likely due to a thinning effect of the IAA treatment, as the plant yield was 5% of the untreated control (Barker and Collins, 1965). A greater number of studies have reported the influence of the synthetic auxin Naphthaleneacetic acid (NAA) on berry size and yield. This may be because NAA has been shown to be more stable, resistant to oxidation, and persist longer in plant tissues compared to IAA (Nissen and Sutter, 1990). The first study on NAA sought to understand the effect of this auxin on NHB plant morphology and berry mass and noted that NAA (0.05 – 500 ppm) increased berry size, while yield was not measured (Mainland and Eck, 1971). One study in RE showed that NAA (10,000 – 50,000 ppm) had an inconsistent effect on berry size and yield (Austin, 1983). More recently (Milić et al., 2018), found that NAA (10 – 20 ppm) increased both berry mass and yield of NHB, and the response was greater with a higher concentration of NAA from 10 to 20 ppm. This study pointed to NAA promoting a greater shoot diameter, and slight increase in shoot length and leaf number per shoot.

ER PGRs have been noted to impact fruit size both directly through the regulation of ripening and indirectly through a thinning effect (Howell et al., 1976; McArtney and Wells, 1995). Mainland and Eck (1971) first applied ethephon (240 to 3840 ppm) to NHB two weeks before harvest (BBCH 89) and noted that concentrations of 1920 ppm and higher caused a reduction in berry mass in the first two (of four) harvests. The change in berry mass was explained by the higher ethephon concentrations both hastening ripening and inducing berry coloration at a smaller berry mass relative to the control. Howell et al. (1976) examined ethephon (500 to 2,500 ppm) as a harvesting aid to reduce retention force for mechanical harvest of NHB and observed that all concentrations of ethephon reduced berry mass significantly in one of two seasons due to stimulating earlier coloration, but did not impact yield. One study in RE did not find an effect of ethephon (200 ppm) on altering berry mass in RE (Ban et al., 2007). More recently, a study in RE revealed that ethephon (250 ppm) applied at 30-40% blue fruit also did not impact berry mass despite an advancement of ripening and increase in berry coloration (Wang et al., 2018). Abscission has been noted as low as 200 ppm, while some cultivars are non-responsive to ethephon until 2000 ppm or higher (Ban et al., 2007; Malladi et al., 2012). Overall, ethylene has a highly variable effect on berry size. Responses to ethylene are genotype-specific, and caution should be made before applying ethephon to blueberry.

Similar to ER PGRs, MeJA at high concentrations (2238 ppm) have been shown to decrease fruit size (Wang et al., 2022). The effects of MeJA on blueberry size have been reported twice in the literature. Percival and MacKenzie, 2007) noted a 35% reduction in berry mass in LB following 10 ppm MeJA treatment when applied in late summer, before berry coloration, approximately BBCH 81. However (Wang et al., 2018), noted no change from control under 112 ppm MeJA treatments in two RE cultivars when applied at the 30-40% ripe fruit stage (BBCH 893-895). The greater concentrations in used by (Wang et al., 2018) compared to (Percival and MacKenzie, 2007) and difference in species used may account for the difference in responses.

One study trialed the effect of MT (11.6 – 116 ppm) on berry mass in NHB (Zheng et al., 2024). MT was applied immediately before berry coloration (BBCH 75 – 78) and no impact on berry size was noted at harvest. This study suggested that berry size was not impacted by MT; however, studies with this PGR are limited.

#### 3.3.3 Ripening rate and harvest fruit quality

Currently, an increasing percentage of blueberries are sold for the fresh market in the U.S. market (USDA/ERS, 2021). The market price for fresh-market blueberries is dictated by the balance between quantity supplied and demanded. The harvest date is an important component of fresh-market production, as the balance between supply and demand for fresh blueberries changes daily during the growing season (Yeh et al., 2023). This is becoming increasingly important as global blueberry acreage continues to rise, complicating supply chain logistics (Arnade and Kuchler, 2015). For this reason, strategies with the capacity to modulate ripening rate and harvest time hold immense economic value.

The harvest quality of blueberries is important for determining value (Canales et al., 2024). Blueberries are commonly sorted based on size, color, and firmness, and can be further graded into premium classes following harvest based on berry size, diameter, and sweetness. While PGRs have been evaluated extensively to alter ripening rate or improve quality traits, there were insufficient observations to conduct a meta-analysis with any one PGR.

Studies evaluating the effect of GA_3_ in blueberry noted a delay in ripening and reduction in total soluble solids (TSS) versus the control (Mainland and Eck, 1971; NeSmith et al., 1995; Williamson et al., 1996). These studies were conducted in RE with the goal of increasing fruit set. Meanwhile, the impacts on fruit quality are variable (Milić et al., 2018; Fujisawa et al., 2019). In RE, few studies measured TSS or berry firmness, most likely due to RE being primarily used for processing where these attributes are less important than berry size or yield. One study in RE measured berry TSS and found that an application of GA_3_ (104 – 485 ppm) at FB reduced the TSS of berries by an average of 18% (Cano-Medrano and Darnell, 1998). In SHB (Fujisawa et al., 2019), noted a reduction of berry TSS of around 7% after 100 ppm treatment between four and six days after FB (BBCH 69). The only NHB trial using GA_3_ did not measure berry TSS, firmness, or any other quality parameter, so there is no consensus on the effect of GA_3_ on highbush blueberry quality. The lack of seeds in GA_3_ pollinated RE berries is thought to contribute to the slower ripening rate (Williamson et al., 1996; NeSmith and Krewer, 1999). Since SHB and NHB berries do not respond to GA_3_ through parthenocarpy under normal pollination conditions, the ripening rate is not impacted{Citation}. However, this difference in response to GA_3_ parthenocarpy between highbush and RE species is not well documented.

CKs have been widely trialed to increase berry size in blueberry, but their influence on ripening rate has received less attention, and only one study examined their impact on fruit quality. Several studies suggested that exogenous CK applications delay ripening, which may impact fruit quality (NeSmith, 2002, 2008). Although several studies recorded ripening rate, techniques used to measure this parameter differed across studies, making a meta-analytic comparison impractical. For example, CPPU (10 ppm) applied at FB in RE delayed ripening and resulted in 25% fewer ripe fruit at the first harvest, and an application two weeks after FB led to 73.8% less ripe fruit at first harvest compared to the control (NeSmith, 2002). In a follow-up study, Nesmith and Adair (2004) observed a similar delay with CPPU (10 ppm) applied shortly after FB, though yield at harvest remained unchanged due to increased berry mass. Since fruit set was doubled in the CPPU treatments, a delay in ripening in RE studies was attributed to the higher crop load. Serri and Hepp (2006) studied the effects of CPPU (10 ppm) on two NHB cultivars grown in Chile and noted that CPPU applied two weeks after FB (BBCH 71) delayed the harvest of a NHB cultivar by two weeks, which was much longer than any RE study. Interestingly, the CPPU application did not affect the harvest date of a second NHB cultivar with a similar ripening time (Bassil et al., 2020). Retamales et al. (2014) trialed repeated post-FB (BBCH 69 – 71) applications of CPPU (5 – 10 ppm) on NHB and discovered that CPPU caused TSS to accumulate more slowly in fruit compared to the control, suggesting a delay in the onset or progression of ripening. Meanwhile, the Blue Color Coverage Index, a measure of the completeness of blue color of fruit at harvest was not impacted. A study conducted in 2016 and 2017 applied CPPU (10 ppm) to a NHB cultivar at FB (BBCH 67) and found only a slight (non-significant) delay in berry maturity across both seasons (Fujisawa et al., 2019). The same study examined the impact of CPPU on fruit quality and noted no change in TSS, which was the only quality metric evaluated. A delay in ripening with applications of CPPU after FB (BBCH 69 – 72) appears across the literature. While application during FB had a lesser effect on ripening rate, effects on fruit quality have largely gone unstudied.

**Figure 11:**
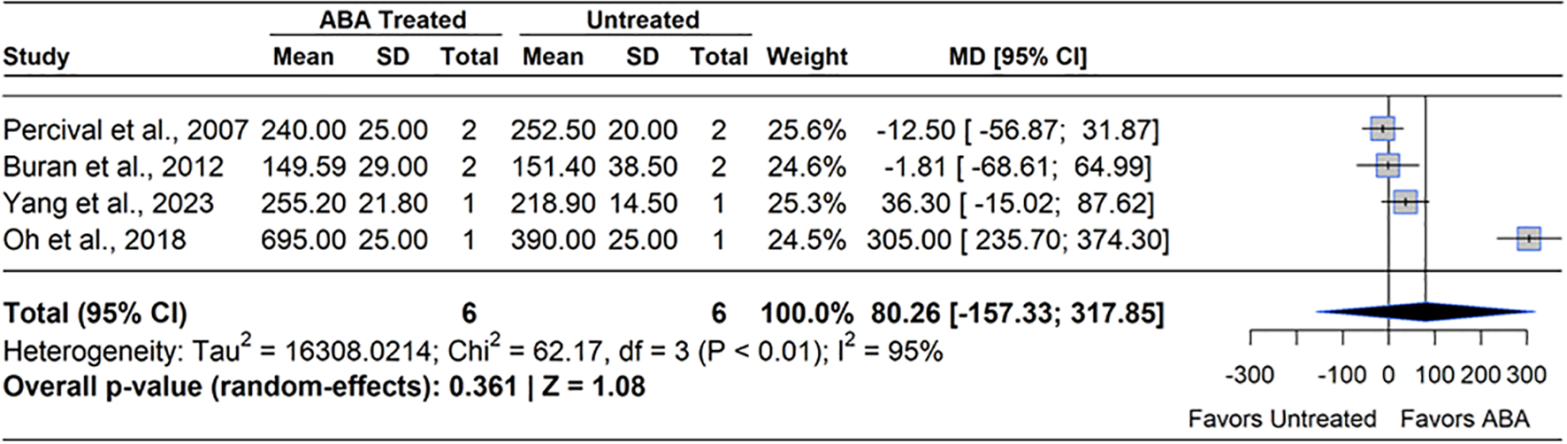
Forest plot of the mean difference (MD) in berry anthocyanin concentration (mg/L) between abscisic acid (ABA)-treated and untreated blueberry plants. Each grey square represents an individual study’s effect size, with square size proportional to the study’s weight. The black diamond represents the overall mean difference, with its width reflecting the 95% confidence interval. The overall effect is considered significant if either value representing the 95% CI does not cross zero. Heterogeneity statistics are presented below the total estimate, and the bolded *p*-value also indicates overall effect.

ABA is known to support ripening and coloration in blueberry through modulation of genes involved in photosynthesis, auxin metabolism, and anthocyanin biosynthesis (Chung et al., 2019; Liu et al., 2022b). ABA levels are known to rise during the onset of ripening, and peak at the ripe stage (BBCH 89) (Zifkin et al., 2012). One study has been conducted to identify the impact of exogenous ABA on ripening rate (Wang et al., 2018). The study applied ABA (600 ppm) at 30 – 40% ripe fruit (BBCH 893) and found no statistical difference in percentage ripe fruit in ABA treated plants and control at any sampling date. The major proxy for blueberry ripeness and harvestability is the dark blue color of fruits, represented by the concentration of anthocyanins in the exocarp. Meta-analysis of four studies on anthocyanin concentration at harvest revealed that ABA does not have a consistent effect of modulating anthocyanin concentration (MD= 32.3%, 95% CI: [-157.33, 317.85], *p*=0.361) (**Figure 11**). Heterogeneity statistics (I²=95%, τ^2^ = 16308.02) indicated extremely high between-study variability (Schwarzer et al., 2015). Among phytohormones, ABA has been researched the most regarding harvest fruit quality. Yet, its effects on fruit quality parameters are inconsistent across studies. For instance (Buran et al., 2012), applied ABA (200 – 400 ppm) to SHB at 75% ripe fruit (BBCH 78) and found no significant differences in berry firmness, anthocyanin or flavanol concentration. Whereas two studies reported increased anthocyanin content and reduced berry firmness following ABA application at higher (528 – 1000 ppm) concentrations (Oh et al., 2018; Zhou et al., 2021). One study observed no significant changes in berry TSS, titratable acidity (TA), or pH with ABA concentrations up to 1000 ppm, beyond which phytotoxic effects appeared. While endogenous ABA levels naturally rise during the transition from pink to blue fruit (Chung et al., 2019), the variability of outcomes in response to ABA application suggest that sensitivity may depend, in part, on surface morphology influencing ABA uptake and translocation (Blumenfeld and Bukovac, 1972; Wan et al., 2024). Previous studies showed that differences in ABA receptor protein or SNRK2 kinase abundance differentiated ABA responsiveness in ripening fruits, highlighting cultivar-specific differences in ABA signaling (Zhao et al., 2019; Liu et al., 2023). It should also be noted that earlier applications of ABA such as shortly after pollination (BBCH 69 – 72) may impact different developmental processes in the fruit, but this has not been evaluated to date.

Auxin concentrations in blueberry peak during seed set and decline during the early stages of fruit ripening (Goto et al., 2013), and a secondary increase in auxin levels has also been observed during the late stages of fruit development in SHB (Liu et al., 2022b). While auxin is not strongly associated with final berry size (Johnson et al., 2011), its accumulation during maturation, along with the expression of specific auxin-responsive genes, suggests a potential role in regulating ripening and fruit quality traits. Only one study noted the impact of auxin applications on blueberry, but this study did not measure any fruit quality parameters (Milić et al., 2018).

ER compounds such as ethephon have been explored as tools to accelerate ripening and improve harvest uniformity in blueberry. Meanwhile, studies on ethylene inhibitors such as 1-Methylcyclopropene (1-MCP) and aminoethoxyvinylglycine (AVG) have largely been limited to post-harvest applications. The effects of ethephon on ripening rate of blueberry generally show advancement in the harvest date. Early studies in the 1970s and 1980s reported that ethephon (500-2500 ppm) applied at BBCH 81 – 89 promoted faster ripening, and increased firmness in some cases (Howell et al., 1976; Dekazos, 1983). Work by (Ban et al., 2007) demonstrated that a RE cultivar experienced an advancement in maturity, but a decrease in berry firmness when a lower concentration (200 ppm) of ethephon was applied at the onset of visible fruit coloration (BBCH 89). Similarly (Wang et al., 2018), applied ethephon (250 ppm) to RE at BBCH 893, when 20 – 40% of berries were already ripe, and observed a significant advancement in fruit ripening rate across two RE cultivars. Notably, this effect was not associated with differences in post-harvest quality, including firmness (Wang and Nambeesan, 2024). expanded ethephon treatment to SHB and saw similar results; ethephon-treated fruit ripened faster, resulting in 35.5% more ripe fruit at five days post-treatment and 17.5% greater ripe fruit at harvest (14 days post-treatment). There have been no recent studies on the influence on pre-harvest ER treatment on ripening or fruit quality of NHB. The relationship between internal ethylene content and berry firmness is of interest to blueberry breeders, and was discussed in a recent study that found that the effects of ethylene on berry firmness are cultivar specific and related to the cultivar’s endogenous ethylene levels during ripening (Farneti et al., 2022). The complete mechanism by which ethylene impacts firmness is still not clear; however (Wang et al., 2020), found that exogenous ethylene promoted cell wall degradation in blueberry through the stimulation of pectin degrading enzymes PE, PG and beta-galactosidase.

To our knowledge, only one study evaluated the impact of MeJA on ripening rate. Wang et al. (2018) applied MeJA (112 and 224 ppm) at the onset of ripening (BBCH 89), but did not observe any difference in ripening rate. Zhang et al. (2017) noted that MeJA (2243 ppm) applied to NHB at BBCH 78 – 79 and 89 increased TSS by 15.6%, signifying an induction of ripening related pathways. MeJA has been shown to stimulate the biosynthesis of secondary metabolites important to quality in fruit crops (VanderWeide et al., 2023), and has been studied for this effect in blueberry. Wang and Nambeesan (2024) found ethylene and jasmonates (MeJA and JA) to be antagonistic, as multiple JA biosynthesis genes were decreased by ethylene application. This suggests that JAs may act as a hormonal signal that promotes the accumulation of anthocyanins and flavor compounds independent from ethylene (Liu et al., 2022a; Wang et al., 2022; Wang and Nambeesan, 2024). Cocetta et al. (2015) noted that MeJA (22 ppm) applied to NHB at BBCH 89 upregulated the expression of *VmPAL*, *VmCHS*, and *VmANS*, which resulted in higher accumulation of delphinidin- and cyanidin-based anthocyanins, as well as increased levels of epicatechin and quercetin flavonoids during the late ripening stages (BBCH 897). Application of 112 and 224 ppm MeJA at the onset of ripening (BBCH 89) did not result in changes to anthocyanin concentration or berry firmness, but caused a transient increase in TA and decrease in berry pH that was not different from the control at harvest (Wang et al., 2018). Applications of MeJA at 4486 ppm have been shown to induce fruit drop in RE and SHB when applied at the ripe fruit stage (BBCH 893) (Malladi et al., 2012). Notably, the observed fruit drop was an intended outcome, as the treatments were applied to promote abscission for harvest facilitation.

Other classes of hormones have become of interest for their potential role in modulating ripening rate of fruit quality only recently, including melatonin (MT). In plants, MT has a role in alleviating abiotic stress conditions like drought (Bidabadi et al., 2020; Khattak et al., 2023), and biotic conditions such as plant pathogens (Tiwari et al., 2022). MT application induced wax formation in blueberry leaves (Shang et al., 2021), which has promise to improve pre- and post-harvest quality. However, the impacts of pre-harvest applications on fruit quality have not been evaluated to date.

#### 3.3.4 Post-harvest fruit quality

Blueberries are often transported great distances to reach the consumer and must maintain quality during shipment (Eum et al., 2013). Various studies have highlighted how PGRs can affect the storability and post-harvest fruit quality of blueberries. However, no hormone has been studied to the depth that a meta-analysis could be conducted. Most studies discussed applied PGRs after berries were harvested rather than pre-harvest, which could reduce the application volume significantly. A challenge to post-harvest PGR application is that wetting blueberries after harvest accelerates post-harvest decay and may spread infection between berries.

Among hormones, ER PGRs were the most studied for their effect on post-harvest fruit quality. Ethylene production generally enhances blueberry fruit ripening, which can paradoxically accelerate post-harvest deterioration and reduce shelf life (Wang et al., 2020; Farneti et al., 2022). In a study in NHB, ethylene gas (10 μl l^-1^) applied to detached ripe berries improved sucrose metabolism and glucose and fructose concentration of blueberries post-harvest storage, but also resulted in a loss of berry firmness via pectin degradation and increased pectinesterase and polygalacturonase activity (Wang et al., 2020). Furthermore, post-harvest ethylene application led to a notable increase in total anthocyanin content, with less consistent modulations to phenolic acid and antioxidant activity. Studies suggest that the application of 1-MCP, which binds to ethylene receptors and inhibits ethylene action, has not shown a consistent ability to extend the shelf life of highbush blueberries and preserve fruit quality attributes (DeLong et al., 2003; Chiabrando and Giacalone, 2011). One study did not find an impact of 1-MCP on the percentage of marketable NHB berries after 12 weeks of storage (DeLong et al., 2003). The same study found no difference in marketable berries even with concentrations of 1-MCP ranging 16-fold, indicating either that 1-MCP saturates the ethylene receptors of blueberry tissue at relatively low concentrations, that ethylene receptor turnover is limiting, or that blueberry fruit may be fundamentally less responsive to ethylene-mediated postharvest deterioration processes than other climacteric or even semi-climacteric fruits. Chiabrando and Giacalone (2011) found that 1-MCP treated NHB berries had significantly less water loss during storage in both years (2006: 12%; 2007: 6.4%); however, this did not impact shelf life. Interestingly (MacLean and Scott NeSmith, 2011), applied 1-MCP to RE and observed a negative effect on fruit firmness during storage, and a significant increase in ethylene in the 1-MCP treated fruit samples. This brings up additional questions surrounding the differences between highbush and RE species and responsiveness to ethylene and antagonists. Dekazos (1979) found that AVG applied to two RE cultivars at 5,000 and 10,000 ppm before FB did not impact berry TSS, pH, TA, or puncture force in ripe fruit, nor did it affect post-harvest storage. The purpose of this study was to protect floral buds from freeze damage, which explains the application at the end of endodormancy, before FB. Watanabe et al. (2021) applied AVG at the green fruit stage (BBCH 75) and noted a reduction in firmness loss after harvest, but no further studies have been conducted using this inhibitor. It should be noted that after ethylene is produced in fruit, AVG will not inhibit its effects, as AVG functions upstream by inhibiting ACC synthase, the enzyme responsible for converting S-adenosylmethionine (SAM) to 1-aminocyclopropane-1-carboxylic acid (ACC), the immediate precursor of ethylene. By preventing further ACC formation, AVG indirectly limits this sustained ethylene signaling, which may explain its efficacy in delaying ripening and reducing tissue softening.

Like ethylene, ABA is also involved in promoting blueberry ripening and could negatively impact shelf life and post-harvest quality. However, several studies indicated that ABA application enhances quality parameters in blueberries during post-harvest storage. This is particularly true for berry color. One study reported that ABA (106 – 212 ppm) applied to detached ripe RE fruit led to a notable increase in anthocyanin concentration after seven days of storage, with higher anthocyanin concentration maintained even after 35 days in storage (Qiao et al., 2024). The authors also indicated that ABA-treated berries had higher TSS at 14 and 21 days in storage. Zhou et al. (2021) also indicated that ABA (528 ppm), applied to detached ripe NHB berries, had a higher TSS during storage, in this case at four- and eight-days post-harvest. ABA was shown to have no impact on TA during storage in (Qiao et al., 2024); however (Zhou et al., 2021), noted that ABA diminished TA at a faster rate than control in storage. This indicates that ABA has capacity to increase TSS in both RE and NHB species post-harvest. One study supported that ABA negatively impacted berry firmness during postharvest storage, by modulating cell wall degrading enzymes. ABA promoted postharvest blueberry softening in (Zhou et al., 2021) by regulating cell wall metabolism and phytohormone accumulation while an ABA inhibitor (nordihydroguaiaretic acid, NDGA) delayed ABA-induced postharvest blueberry softening. The increase in softening occurred due to ABA reducing non-soluble pectin, cellulose, and hemicellulose, and increasing pectinesterase, polygalacturonase, and β-galactosidase enzyme activity, as well as the expression of genes encoding these enzymes. Meanwhile, the only other study indicated only a minor impact on firmness during post-harvest storage (Qiao et al., 2024). Interestingly, the same study showed the capacity of ABA to maintain aroma concentrations in berries during storage (Qiao et al., 2024), and even increased ester concentrations such as hexyl propionate and ethyl 2-methylbutyrate, which contribute to fruit flavor (Gilbert et al., 2015).

CK was trialed once for improving post-harvest fruit quality. Post-FB applications of CPPU (510 ppm) were applied to NHB (Retamales et al., 2014). The greatest difference in fruit quality by CPPU treatments was a significant reduction in the loss in berry mass during storage. Some treatments with high concentrations or multiple applications of CPPU reduced TSS at harvest, but no differences were observed after four weeks of post-harvest storage.

MeJA is a plant signaling molecule that has gained interest for its potential to enhance the shelf life of various fruit, including blueberries (González-Aguilar et al., 2000; Saavedra et al., 2016; Wang et al., 2021). Studies indicate that MeJA application significantly affected fruit quality attributes that are critical for maintaining shelf life, including resistance to pathogens and cold injury (González-Aguilar et al., 2000; Yao and Tian, 2005; Meng et al., 2009; García-Pastor et al., 2020). One study showed that MeJA (11 and 22 ppm), applied to detached ripe RE berries, delayed blueberry softening during storage by reducing the activity and expression level of enzymes relating to cell wall degradation (Wang et al., 2019). Wang et al. (2021) applied MeJA to RE at 11 ppm post-harvest and observed MeJA treated berries had higher firmness throughout post-harvest storage.

MT is an understudied phytohormone in blueberry; only a few studies have revealed a promising effect on post-harvest fruit quality. Pre-harvest application of MT (5 ppm) applied to NHB at 50% ripe fruit (BBCH 75) significantly reduced water loss in post-harvest storage. Treatments also maintained TA and increased total phenolics, flavonoids, and anthocyanins, but also had a negative impact on TSS during storage (Zheng et al., 2024). Maintenance of fruit quality may be related to a close connection with antioxidant pathways. MT has been observed to decrease the activity of polyphenol oxidase (PPO), a key enzyme involved in browning and deterioration in fruit, by modulating antioxidant systems (Khan et al., 2020). Its application has been shown to enhance antioxidant metabolism, reduce oxidative stress, and ultimately minimize post-harvest losses. MT has been shown to suppress ethylene production in other fruit crops, which may preserve fruit quality, but this has not been studied in blueberry. MT may work to reduce the softening and senescent properties of ethylene evolution in post-harvest.

#### 3.3.5 Winter hardiness

Cold stress is a major threat to blueberry production, regardless of the production environment. In the northern U.S. and Canada, NHB and LB are exposed to low winter temperatures during endodormancy. Furthermore, these species are susceptible to frost events in the fall, but particularly in spring as plants acclimate and subsequently de-acclimate from endodormancy. Early spring frost events are also a common occurrence in the Southeastern U.S., which can cause injury to SHB and RE cultivars (NeSmith et al., 1995). Spring freeze events have become more common over the past decade across the U.S. due to warming spring temperatures as a result of climate change (Wang et al., 2024). This causes earlier bud development when there remains risk for frost events. Floral buds, followed by vegetative buds, represent the most susceptible tissues to cold stress (Ehlenfeldt et al., 2012). Since blueberry fruit buds form in the season prior, damage to floral buds diminishes yields for the following growing season.

Changes to endogenous hormone concentrations occur in fruit buds during the transition to and release from dormancy. After 600 chilling hours were reached, ABA increased and IAA decreased in RE floral bud tissue until 900 chilling hours were reached, and then declined and increased, respectively (Lin and Agehara, 2021). In SHB, ABA, SA, and ACC contents decreased sharply at the release of eco- and endodormancy, while JA decreased and then increased at both stages, respectively (Liu et al., 2022a). These patterns suggest that the dynamic balance between IAA and ABA, rather than absolute levels alone, may serve as a regulatory cue for dormancy progression and release.

The use of PGRs to improve cold hardiness and mitigate freezing stress may be limited by the fact that applying PGRs to dormant plants during the winter is uncommon, mainly due to poor field accessibility and availability of equipment and labor. Additionally, hormone absorption would be limited by the potential presence of snow or ice on bud tissues in some growing regions and dry air conditions in others. For this reason, the use of PGRs to improve winter hardiness may be more promising in SHB and RE production regions. Few studies have evaluated the potential of PGRs to delay FB or improve hardiness, which would reduce the risk of freeze injury on flower buds. In RE, a pre-FB (BBCH 51) application of 1000 ppm ABA applied to whole plants only delayed FB by one day compared to an untreated control (Sams et al., 2010). However (Panicker and Matta, 2016), showed that ABA concentrations as low as 20 ppm applied to detached RE flowers were able to induce higher cold hardiness, for at least the first flush of floral growth, showing that ABA is an important factor in cold hardiness, even when the phenology of floral development is not impacted. Dekazos (1979) reported that applications of 5,000 and 10,000 ppm AVG to RE at five weeks before FB delayed FB by 10 days. This length of a delay in FB could help flowers to avoid a damaging spring frost event.

2,4-Epibrassinolide (EBR) has been shown to reduce the extent of membrane lipid peroxidation in response to cold temperatures, a critical component of cold hardiness (Chen et al., 2022). One study tested the effects of EBR on blueberry cold hardiness; (Fan et al., 2025) applied low concentrations of EBR (0.2 – 0.8 ppm) on expanding flower buds of NHB then induced chilling events, and noted that osmotic substances classified as ‘solid proteins,’ ‘soluble solids,’ and ‘proline’ were induced under EBR treatment. The study also noted EBR application induced the antioxidant enzymes SOD, POD, and CAT, which led to treated buds accumulating less ROS. Genetic analysis of *VcCBF3*, a gene recently found to have importance in regulating cold tolerance (Walworth and Song, 2018), was found to be greatly upregulated under EBR application, as high as 365% greater than control (Fan et al., 2025).

### 3.4 Factors influencing phytohormone absorption into plant tissues

#### 3.4.1 Cuticle and stomata

To impact plant growth and development, the active ingredient (AI) of a PGR must enter epidermal cells. This requires that the AI crosses the cuticle or through stomatal pores. The cuticle is a waxy boundary layer made up of lipid-derived components that coats epidermal cells lining the aerial organs of vascular plants, including leaves and fruit. The cuticle’s unique chemical properties allow gases and small amounts of water and nutrients to enter and leave epidermal cells while maintaining organ structure. The cuticle is made up of two distinct layers: the cuticle layer, containing embedded polysaccharides, and the cuticle proper, which contains mainly cutin and waxes (Bargel et al., 2006). The fruit cuticle’s main functions are to prevent water loss from various tissues and protect the plant from abiotic and biotic environmental stressors (VanderWeide et al., 2022b; Yan and Castellarin, 2022). The cuticle is the direct physical interface between the fruit and the external environment. Over the season, the biosynthesis of cuticle components is continuous, but the cuticle may develop microcracks or physical damage that facilitate the greater entry of solutes. Transportation through the cuticle is controlled by diffusion and is classified into three processes: (1) sorption into the cuticle, (2) diffusion through the cuticle, and (3) desorption from the cuticle (Bukovac et al., 1990; Kirkwood, 1999). First, sorption into the cuticle involves the initial interaction of the hormone with the cuticle. Sorption is linear for some hormones, and multiphasic for others (Blumenfeld and Bukovac, 1972; Bukovac and Petracek, 1993; Schönherr et al., 2000a). It is also dependent on the cuticle’s adhesive properties and surface topography. Second, diffusion through the cuticle entails interactions between the hormone and lipids in the cuticle layer. Lipophilic hormones travel faster through the cuticle than hydrophilic molecules (**Supplementary Table S1**). Finally, desorption from the cuticle refers to a hormone moving through the cuticle into epidermal cells, either through the apoplast or vascular tissue.

Evidence supports that aqueous solutes can pass through open stomatal pores if there is a positive pressure gradient (Schönherr et al., 2000a). A positive pressure gradient occurs when the pressure outside the leaf surface, such as from a spray droplet or surface tension, exceeds the internal pressure in the substomatal cavity, allowing solutes to be physically forced into the pores. This pressure-driven flow through a porous opening can be described by Darcy’s Law, which relates flow rate to pressure difference, fluid viscosity, and pore characteristics (Hillel et al., 1998). This phenomenon has been illustrated in studies describing the penetration of auxins as NAA and IAA, ethylene as ethephon, gibberellic acids as GA_3_ and GA_4 + 7_, jasmonates as Methyl Jasmonate, cytokinins as 6-BA and CPPU, and abscisic acid as s-ABA through stomatal pores (Blumenfeld and Bukovac, 1972; Bukovac et al., 1990; Knoche and Bukovac, 1991; Ben-Tal and Wodner, 1993). In all cases, uptake of the hormone was rapid compared to uptake though the cuticle, irrespective of the amount taken up by epidermal or mesophyll tissue. Solutes penetrate through the stomata by diffusion through the thinner, inner liners of the guard cells in a manor dependent on the aperture size.

#### 3.4.2 Adjuvants

Water is the carrying agent for PGR applications, but does not adhere well to the cuticle of plant tissues. Additionally, the phytohormone AI of most PGRs is not hydrophilic, as indicated by their high LogP or partition coefficient values (Mackay et al., 2006). The need for surfactants and the efficacy of foliar PGR applications differs substantially by species, in part due to variation in cuticle properties and the physicochemical characteristics of the PGRs themselves. Hydrophobic compounds, such as BR and jasmonates (LogP > 2), require oil-based or nonionic surfactants to cross lipophilic barriers, whereas hydrophilic molecules like ethephon (LogP ≈ -1.0) can be absorbed more readily in aqueous formulations. Inert compounds called “adjuvants” are therefore required to improve phytohormone miscibility in water, as well as PGR adherence to plant tissues (Bukovac and Petracek, 1993). The droplet contact angle, defined as the angle formed between the surface and the tangent at the edge of the droplet is reduced by the use of surfactant-based adjuvants, which lower surface tension, allowing the droplet to spread more over the leaf surface (Da Silva Santos et al., 2021). This increases the wetted surface area and enhances penetration and absorption of active ingredients. Beyond improving contact angle and penetration, adjuvants have other modes of action, summarized in **Supplementary Table S2**.

The effectiveness of an adjuvant depends on several factors, including the physicochemical properties of the PGR, environmental conditions at the time of application, and the specific characteristics and purpose of application of the target crop (Walters and Lopez, 2018). For example, in blueberries, the presence of a thick wax layer on leaves and fruit can make penetration particularly challenging (Yang et al., 2020), necessitating the use of highly effective penetrants or surfactant systems. Additionally, adjuvant-PGR interactions can influence uptake rates and biological activity, meaning that careful selection of adjuvants is crucial for optimizing treatment efficacy (Stevens, 1993). The thickness of cuticles changes during the season (Yang et al., 2020; Moggia et al., 2022), so adjuvants may assist penetration into plant tissues better during different times of the season. Despite their importance, adjuvants must be carefully evaluated to avoid potential phytotoxicity, unintended effects on fruit quality, or compatibility issues with other agrochemicals in the spray mix (Stevens, 1993).

### 3.5 Conclusion

Blueberry has rapidly emerged as one of the most economically important fruit crops in the U.S. This expansion is mirrored globally and brings with it new challenges in maintaining consistent yield, fruit quality, and shelf life under increasingly variable environmental conditions. Despite the critical role of PGRs in other fruit systems, blueberry remains underserved in both research and regulation. Few PGRs are currently registered for use in blueberry, and the literature is fragmented across species, cultivars, and experimental conditions. In this meta-analysis, we categorized 48 studies by blueberry species, PGR active ingredient (phytohormone), and production topic. The majority of PGR research has focused on CKs and GA_3_, with fewer studies exploring auxins, ABA, or ER, and very few studies examining other phytohormones such as JAs, MT, or BR. Among production traits, “fruit set,” “berry size and plant yield,” and “ripening rate and harvest fruit quality” dominate the literature, reflecting producer priorities around yield and marketability. We identified clear gaps in knowledge that should guide future research: 1) establishing multi-site trials with standardized PGR protocols to evaluate macroclimate effects; 2) testing combinatorial PGR treatments to identify synergistic or antagonistic effects on production outcomes; 3) expanding evaluation of post-harvest fruit quality and shelf-life in PGR trials; and 4) integrating digital decision tools (e.g., ripening models, real-time stress detection) to guide precision PGR applications. As blueberry production moves toward the use of new cultivars, harvest mechanization, and faces intensifying climate pressures, the strategic application of PGRs may offer a lever to improve crop outcomes. Future work must bridge hormone physiology with cultivar-specific responses and real-world production constraints. Unlocking the full potential of PGRs in blueberry will require expanded trials and registrations but also a systems-level understanding of how these tools interact with genotype, environment, and grower management.

## Data Availability

The original contributions presented in the study are included in the article/**Supplementary Material**. Further inquiries can be directed to the corresponding author.

## Supplementary material

Supplementary Figure 1 PRISMA diagram illustrating the study identification, screening, and inclusion process, including the total number of publications identified from databases, screened, excluded, and included in the final analysis.

Supplementary Table 1 Physicochemical classification of common plant growth regulators (PGRs) based on their partition coefficient (LogP), hydrophobicity, and representative PubChem compound identifiers.

Supplementary Table 2 Types and functions of agricultural adjuvants.
