# Supplementary figures and images for: A meta-analysis and systematic review of plant growth regulator use in blueberry production
Source: Front Plant Sci. 2025 Aug 20;16:1632855. doi: 10.3389/fpls.2025.1632855 (PMC12405187; doi:10.3389/fpls.2025.1632855)

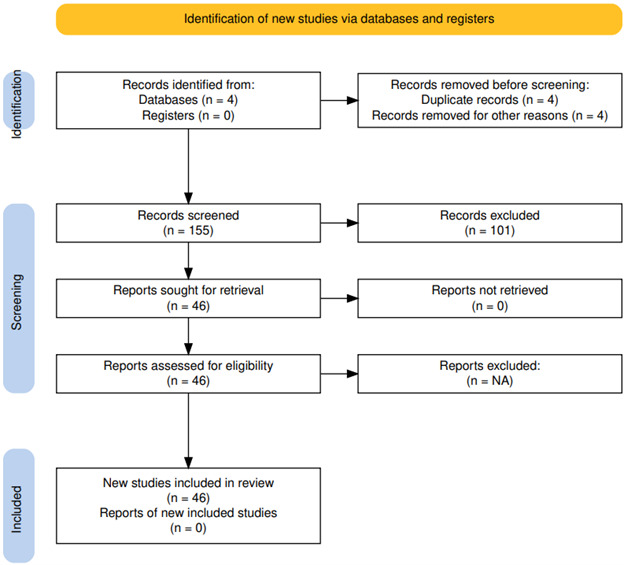

Supplement: Supplementary Figure 1 — PRISMA diagram illustrating the study identification, screening, and inclusion process, including the total number of publications identified from databases, screened, excluded, and included in the final analysis. [file Image1.jpeg]
