# Supplementary material for: A meta-analysis and systematic review of plant growth regulator use in blueberry production
Source: Front Plant Sci. 2025 Aug 20;16:1632855. doi: 10.3389/fpls.2025.1632855 (PMC12405187; doi:10.3389/fpls.2025.1632855)
Supplement: Supplementary Table 2 — Types and functions of agricultural adjuvants. [file Table2.docx]

**Supplemental Table 2.** Types and functions of agricultural adjuvants.

| **Adjuvant type** | **Function** |
| --- | --- |
| Surfactant | Reduce the surface tension of water, improving coverage and penetration of the active ingredient. |
| Emulsifier | Allow oil-based products to mix efficiently with water. |
| Drift Control Agent | Reduce the drift of products during application, improving efficacy and reducing environmental impact. |
| pH Adjuster | Adjust the pH of the spray solution to ensure optimal performance of the product. |
| Antifoaming Agent | Prevent the formation of foam during mixing and application of products. |
| Wetting Agent | Improve the wetting and spreading of the product solution on plant surfaces. |
| Compatibility Agent | Ensure the compatibility of the product and adjuvant when mixed together. |
| Tank Cleaner | Clean the spray equipment and remove any product residues. |
